# Effect of alkaline precipitation chemistry on morphology, colloidal stability, and antibacterial activity of ZnO nanoparticles synthesized using plant extracts

**DOI:** 10.3762/bjnano.17.99

**Published:** 2026-10-21

**Authors:** Fanny Jauregui González, Dora María Frías Márquez, Susana de la Rosa García, Cinthia García Mendoza, Rosendo López González, Mayra A Álvarez Lemus, Sergio Gómez Cornelio

**Affiliations:** 1 Nanotechnology Laboratory-CICTAT, Academic Division of Engineering and Architecture, Juarez Autonomous University of Tabasco, Cunduacan, Tabasco, Mexico; 2 Biotechnology Laboratory, Polytechnic University of the Center, Centro, Mexico; 3 Applied Microbiology Laboratory, Academic Division of Biological Sciences, Juarez Autonomous University of Tabasco, Villahermosa, Mexico

**Keywords:** alkaline precipitating agent, antibacterial activity, physicochemical properties, plant-mediated synthesis, ZnO nanoparticles

## Abstract

The antibacterial performance of plant-mediated ZnO nanoparticles frequently varies despite their similar crystal structure, suggesting that additional synthesis parameters influence their nano–bio interactions. In particular, the influence of alkaline precipitation chemistry on colloidal organization and biological activity remains poorly resolved. In this study, ZnO nanoparticles were synthesized using aqueous leaf extracts of *Piper auritum* and *Mimosa pudica* with NaOH or NH_4_OH as alkaline precipitating agents to evaluate their role in controlling physicochemical and antibacterial properties. Four materials were obtained, designated ZnO-PA, ZnO-MA, ZnO-PS, and ZnO-MS. FTIR analysis confirmed Zn–O formation and the presence of residual phytochemical functional groups on the nanoparticle surface. XRD confirmed the formation of the hexagonal wurtzite phase with crystallite sizes ranging from 15.4 to 25.5 nm. The precipitating agent strongly influenced nanoparticle morphology and colloidal behavior. NH_4_OH produced rod-like particles, whereas NaOH generated quasi-spherical structures. Consistently, ZnO-PS and ZnO-MS exhibited markedly smaller hydrodynamic diameters (214.8–271.5 nm) than ZnO-PA and ZnO-MA (1448–1763 nm), together with more negative zeta potentials and lower polydispersity indices. Antibacterial activity, evaluated by broth microdilution against *Staphylococcus aureus*, *Listeria innocua*, *Escherichia coli*, and *Salmonella typhimurium*, showed strain-dependent activity, with minimum inhibitory concentration (MIC) and minimum bactericidal concentration (MBC) values ranging from 156 to 5,000 µg·mL^−1^ and from 625 to more than 20,000 µg·mL^−1^, respectively. ZnO-PA exhibited bactericidal activity (MBC/MIC ≤ 4) against all tested strains. Overall, these results indicate that alkaline precipitation chemistry governs the colloidal organization and morphology of plant-mediated ZnO nanoparticles, thereby contributing substantially to the antibacterial performance of the resulting nanoparticles.

## Introduction

Zinc oxide (ZnO) nanoparticles have attracted considerable attention as antimicrobial nanomaterials because of their physicochemical versatility and broad-spectrum biological activity [1–2]. Zinc-based nanomaterials are of particular biological interest since Zn^2+^ can actively participate in microbial toxicity pathways through membrane destabilization, intracellular metal ion imbalance, and redox-associated cellular disruption [2]. These metal-mediated effects are strongly influenced by nanoparticle morphology, surface charge, size distribution, and colloidal organization, all of which determine the interaction of zinc-based nanomaterials with microbial cells and ultimately their biological response [2–3]. Consequently, changes introduced during nanoparticle synthesis may directly influence the antimicrobial performance of ZnO through their effects on these physicochemical properties. Owing to these properties, ZnO nanomaterials have found applications across pharmaceutical and biomedical sciences, electronics, cosmetics, and the food industry [4–6].

Among these applications, their antibacterial activity has been extensively documented against a wide range of pathogenic bacteria, including *Yersinia enterocolitica, Pseudomonas aeruginosa*, *Salmonella typhimurium*, *Escherichia coli*, and *Staphylococcus aureus* [4]. The antimicrobial response has been mainly associated with metal-ion-mediated oxidative stress and the generation of reactive oxygen species (ROS), which can alter membrane integrity, interfere with cellular metabolism, and ultimately compromise cell viability [5–7]. However, despite extensive studies on plant-assisted synthesis routes, the influence of alkaline precipitating agents on the physicochemical properties and antibacterial performance of ZnO nanoparticles remains insufficiently understood. Most studies have focused on the role of plant extracts as reducing and stabilizing agents, whereas comparatively little attention has been paid to the influence of precipitation chemistry on nanoparticle formation and the resulting structural and colloidal characteristics. Consequently, it remains unclear whether changing the alkaline precipitating agent is sufficient to modify nanoparticle organization and, ultimately, antibacterial performance.

ZnO nanoparticles can be synthesized through various approaches, including physical, chemical, and biological methods. Physical and chemical synthesis routes are well established and reproducible at the industrial scale; however, they often involve elevated temperatures, specialized equipment, and the use of hazardous solvents or additional chemical reagents that may increase process complexity and environmental impact [8–9]. In contrast, biological synthesis has emerged as a promising alternative due to its relatively mild conditions and the use of renewable biological resources. In these systems, microorganisms or plant extracts provide biomolecules capable of interacting with metal precursors and stabilizing the resulting nanostructures [10–11]. The chemical complexity of plant extracts, particularly their abundance of phenolic compounds, flavonoids, and terpenoid metabolites, provides multiple functional groups capable of interacting with Zn precursors during nucleation, growth, and stabilization processes [12]. Consequently, differences in phytochemical composition may influence nanoparticle formation, morphology, and surface functionalization, thereby contributing to the final characteristics of the resulting nanomaterials.

Although NaOH is the precipitating agent most commonly employed in plant-mediated ZnO synthesis, comparatively few studies have evaluated NH_4_OH [13–15]. This aspect is particularly relevant because the precipitating agent controls hydroxide ion availability, thereby influencing nucleation kinetics and crystal growth during ZnO formation. Differences in hydroxide availability may modify crystal growth, particle aggregation, colloidal stability, and surface characteristics, all of which have been associated with the antibacterial performance of ZnO nanoparticles [14]. However, despite its potential importance, the contribution of precipitation chemistry to these properties has received considerably less attention than that of the biological extract itself. A better understanding of this relationship is therefore necessary to establish how synthesis conditions determine the physicochemical properties and antibacterial activity of plant-mediated ZnO nanoparticles.

Here, we investigated the influence of alkaline precipitation chemistry on the structural, colloidal, and antibacterial properties of biosynthesized ZnO nanoparticles. To address this question, ZnO nanoparticles were synthesized using aqueous extracts of *Piper auritum* and *Mimosa pudica* in combination with two alkaline precipitating agents (NaOH and NH_4_OH). These plant species were selected because of their high content of bioactive secondary metabolites, including polyphenols, flavonoids, and tannins, which can act as reducing and stabilizing agents during nanoparticle formation [16–17]. By comparing two plant extracts synthesized under identical experimental conditions while varying only the precipitating agent, this experimental design enabled the contribution of precipitation chemistry to be distinguished from that of the biological extract. The resulting nanoparticles were characterized by Fourier-transform infrared spectroscopy (FTIR), dynamic light scattering (DLS), electrophoretic light scattering (ELS), field-emission scanning electron microscopy (FESEM), and X-ray diffraction (XRD). Their antibacterial activity was evaluated using a broth microdilution assay against *Listeria innocua* (ATCC 33090), *Staphylococcus aureus* (ATCC 6538), *Escherichia coli* (ATCC 25922), and *Salmonella typhimurium* (ATCC 14028), allowing the relationship between precipitation chemistry, nanoparticle characteristics, and antibacterial performance to be established.

## Results

### Characterization of ZnO nanoparticles

**Figure 1:**
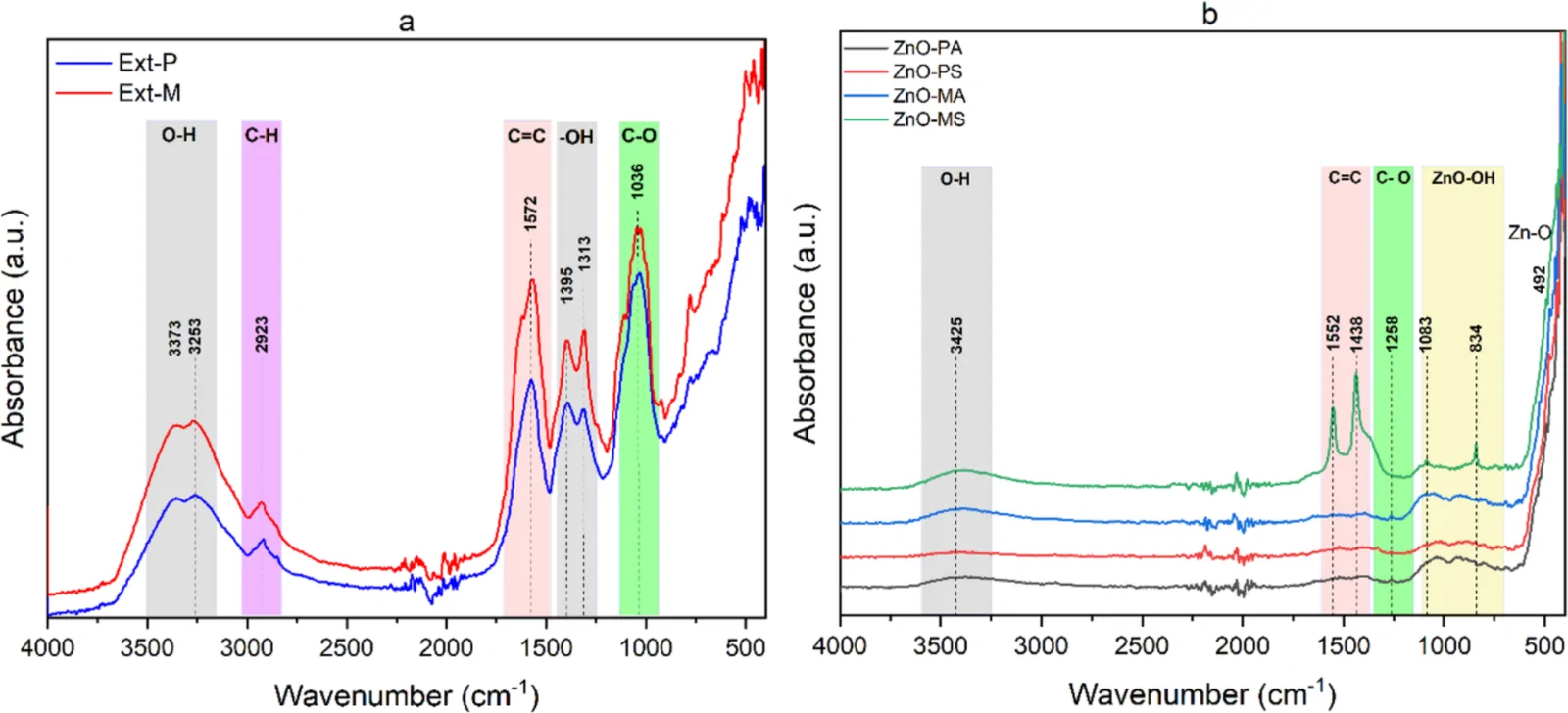
FTIR spectra of (a) the lyophilized aqueous extracts of *P. auritum* (Ext-P) and *M. pudica* (Ext-M) and (b) the corresponding ZnO nanoparticles after calcination at 400 °C.

The synthesized ZnO nanoparticles were designated according to the plant extract and alkaline precipitating agent used during synthesis, that is, ZnO-PA (*P. auritum*/NH_4_OH), ZnO-PS (*P. auritum*/NaOH), ZnO-MA (*M. pudica*/NH_4_OH), and ZnO-MS (*M. pudica*/NaOH). The FTIR spectra of the lyophilized aqueous extracts of *P. auritum* (Ext-P) and *M. pudica* (Ext-M) are shown in Figure 1a. Both extracts exhibited absorption bands at 3373, 3253, 2923, 1572, 1395, 1313, and 1036 cm^−1^. The broad absorptions at 3373–3253 cm^−1^ can be attributed to O–H stretching vibrations, whereas the band at 2923 cm^−1^ is attributed to aliphatic C–H stretching vibrations. The bands at 1572, 1395–1313, and 1036 cm^−1^ were assigned to aromatic C=C stretching, vibrations associated with oxygen-containing functional groups, and C–O-stretching vibrations, respectively. The band positions were similar in both extracts; however, Ext-M exhibited higher absorbance intensities, particularly in the regions around 1572, 1395–1313, and 1036 cm^−1^.

The FTIR spectra of the synthesized ZnO nanoparticles are shown in Figure 1b. All samples exhibited a characteristic band located at 492 cm^−1^, which was assigned to Zn–O stretching vibrations, confirming the formation of the ZnO phase. Additional weak absorption bands were observed at 3425, 1552–1438, 1258, and 1083–834 cm^−1^. The band at 3425 cm^−1^ can be attributed to O–H stretching vibrations, whereas the signals at 1552 and 1438 cm^−1^ and at 1258 cm^−1^ were assigned to C=C and C–O stretching vibrations, respectively. The bands at 1083 and 834 cm^−1^ may be associated with Zn–O–H related vibrations. Minor differences in band intensity were observed among the synthesized nanoparticles, with slightly higher intensities for ZnO-MA and ZnO-MS than for ZnO-PA and ZnO-PS.

**Figure 2:**
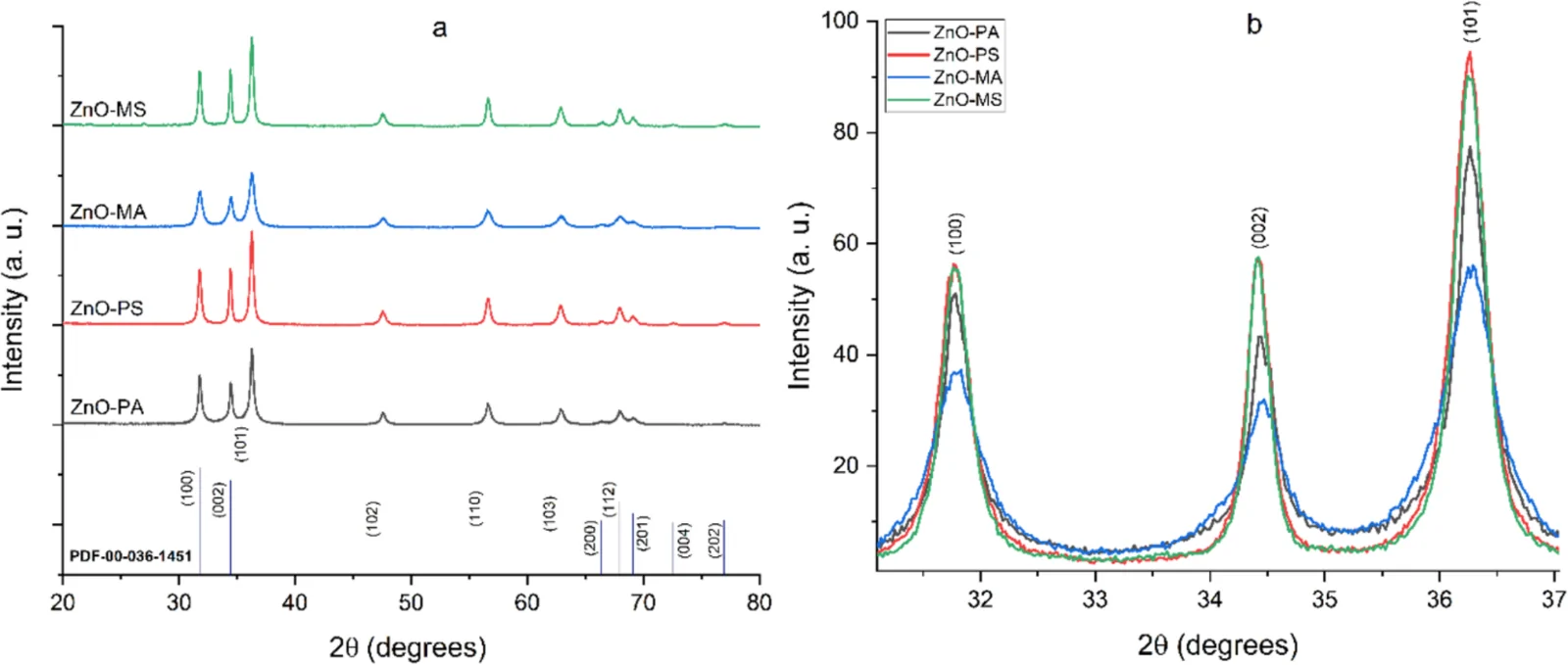
X-ray diffraction patterns of the synthesized ZnO nanoparticles: (a) complete diffractograms and (b) enlarged view of the (100), (002), and (101) reflections.

The XRD patterns of the synthesized ZnO nanoparticles (Figure 2a) exhibited diffraction peaks at 2θ values of approximately 31°, 34°, 36°, 47°, 56°, 62°, 66°, 68°, and 69°, corresponding to the (100), (002), (101), (102), (110), (103), (200), (112), and (201) crystallographic planes of hexagonal ZnO, respectively. The diffraction patterns agreed well with the standard reference pattern of hexagonal wurtzite ZnO (ICDD PDF no. 36-1451). No additional crystalline phases were detected within the resolution of the XRD analysis. Differences in diffraction peak intensity and width were observed among the samples. ZnO-PS and ZnO-MS exhibited narrower and more intense reflections than ZnO-PA and ZnO-MA, whereas ZnO-MA showed the broadest diffraction peaks, particularly for the (101) reflection (Figure 2b).

The crystallite sizes calculated using the Scherrer equation ranged from 15.35 to 25.49 nm (Table 1). ZnO-MA exhibited the smallest crystallite size (15.35 nm), whereas ZnO-PA, ZnO-PS, and ZnO-MS showed similar values, ranging from 24.48 to 25.49 nm. To further evaluate diffraction peak broadening, crystallite size, microstrain, and dislocation density were determined using the Williamson–Hall (W–H) method. The lattice parameters calculated for all nanoparticles were *a* = *b* = 0.325 nm, whereas the *c* parameter was 0.520 nm for ZnO-PA and ZnO-MA and 0.521 nm for ZnO-PS and ZnO-MS. The corresponding Zn–O bond lengths varied only slightly, ranging from 0.1976 to 0.1978 nm. The W–H crystallite sizes followed the same general trend as those estimated using the Scherrer equation, with ZnO-MA presenting the smallest crystallite size by both methods (Table 1). The calculated microstrain values ranged from 1.4 × 10^–3^ to 1.82 × 10^−3^. The dislocation density ranged from 1.19 × 10^15^ to 5.28 × 10^15^ m^−2^, with ZnO-MA exhibiting the highest value (Table 1).

**Table 1:** Crystallite size, microstrain, and dislocation density of the synthesized ZnO nanoparticles determined from XRD analysis.

Sample	Scherrer *D* (nm)	Williamson–Hall *D* (nm)	Microstrain ε	Dislocation density δ (m^–2^)

ZnO-PA	24.48	25.30	1.82 × 10^−3^	1.56 × 10^15^
ZnO-PS	24.50	28.71	1.73 × 10^−3^	1.21 × 10^15^
ZnO-MA	15.35	13.76	1.40 × 10^−3^	5.28 × 10^15^
ZnO-MS	25.49	28.89	1.64 × 10^−3^	1.19 × 10^15^

**Figure 3:**
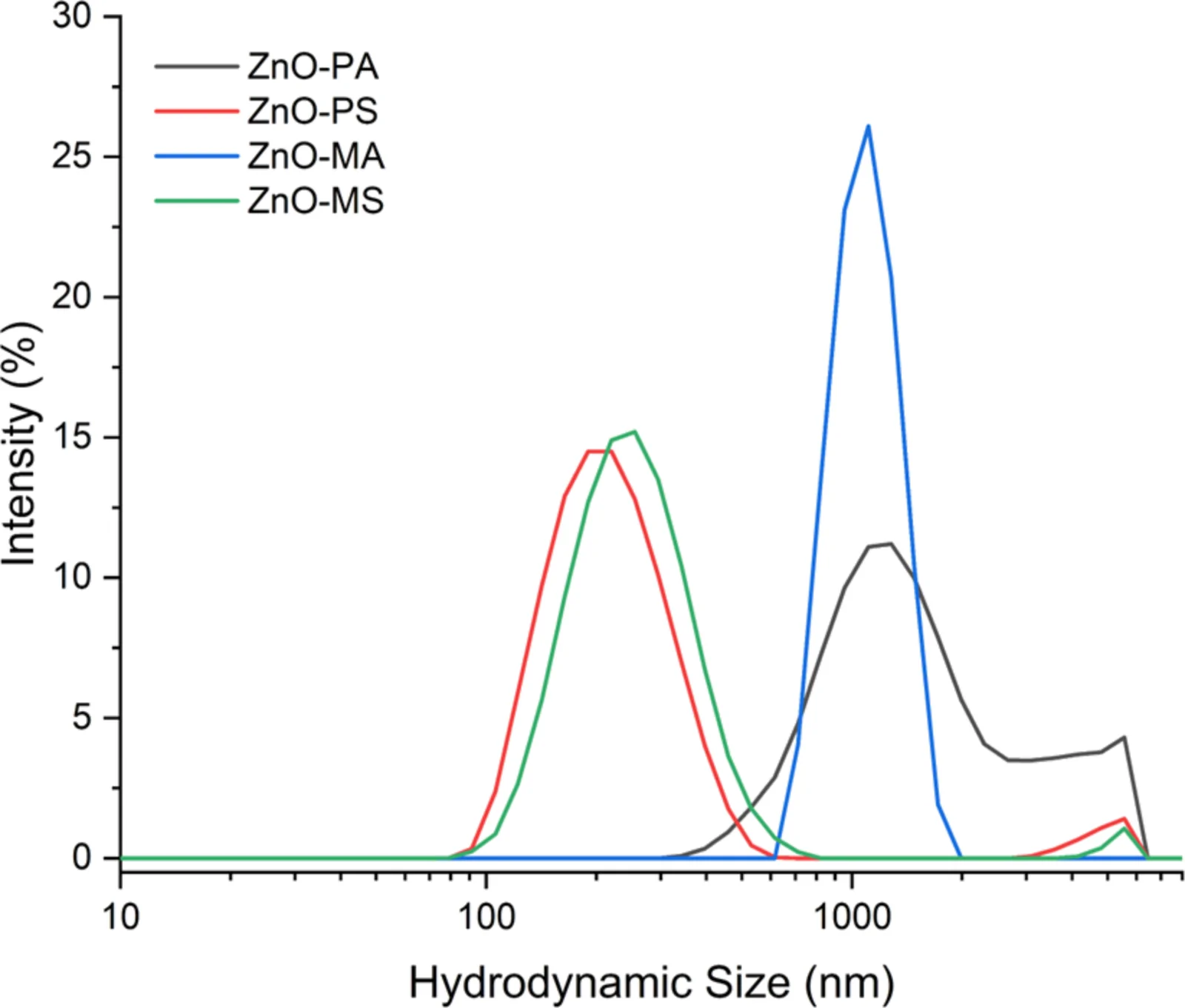
Hydrodynamic size distributions of the synthesized ZnO nanoparticles determined by DLS.

The synthesized ZnO nanoparticles exhibited distinct hydrodynamic size distributions depending on the alkaline precipitating agent used during synthesis (Figure 3). ZnO-PA and ZnO-MA exhibited broad hydrodynamic size distributions, indicating the presence of large aggregates in aqueous suspension. ZnO-PA displayed a broad distribution extending from approximately 100 to 6000 nm, whereas ZnO-MA exhibited a size distribution centered at approximately 1300 nm. In contrast, ZnO-PS and ZnO-MS exhibited considerably narrower distributions centered between approximately 100 and 150 nm.

Average hydrodynamic size (*Z*-average), polydispersity index (PDI), and zeta potential (ζ) are summarized in Table 2. ZnO-PS and ZnO-MS exhibited the smallest average hydrodynamic sizes, with *Z*-average values of 214.8 and 271.5 nm, respectively, whereas ZnO-PA and ZnO-MA presented substantially larger values of 1448 and 1763 nm, respectively. Consistent differences were observed for the PDI values. ZnO-PS and ZnO-MS exhibited PDI values of 0.21 and 0.30, respectively, which were approximately 1.5–1.8-fold lower than those obtained for ZnO-PA (0.38) and ZnO-MA (0.45). The zeta potential varied among the synthesized nanoparticles. ZnO-MS exhibited the most negative zeta potential (−26.3 mV), followed by ZnO-MA (−21.4 mV), ZnO-PS (−15.6 mV), and ZnO-PA (−9.4 mV). Within each plant extract, the nanoparticles synthesized using NaOH exhibited more negative zeta potential values than those synthesized using NH_4_OH.

**Table 2:** Hydrodynamic size (*Z*-average), polydispersity index (PDI), and zeta potential (ζ) of the synthesized ZnO nanoparticles.

Sample	*Z*-average (nm)	PDI	ζ (mV)

ZnO-PA	1448.0	0.38	−9.4
ZnO-PS	214.8	0.21	−15.6
ZnO-MA	1763.0	0.45	−21.4
ZnO-MS	271.5	0.30	−26.3

**Figure 4:**
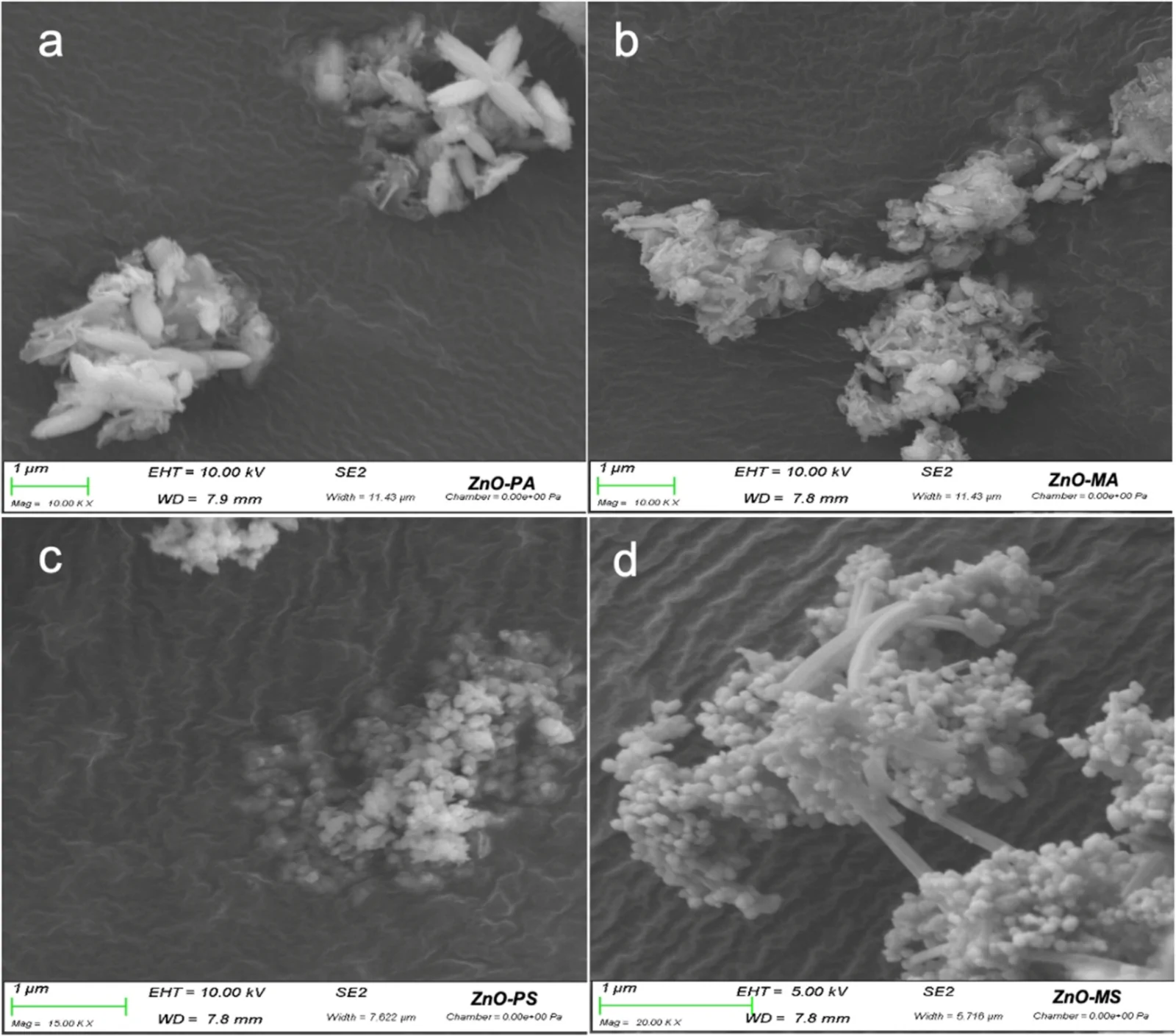
FESEM micrographs of the synthesized ZnO nanoparticles: (a) ZnO-PA, (b) ZnO-MA, (c) ZnO-PS, and (d) ZnO-MS.

SEM analysis revealed distinct particle morphologies among the synthesized ZnO nanoparticles (Figure 4). ZnO-PA and ZnO-MA formed aggregates of predominantly rod-shaped particles (Figure 4a,b). ZnO-PA exhibited the largest rod-shaped particles, with an average width of 288.27 ± 52.69 nm and an average length of 780.74 ± 122.29 nm, whereas ZnO-MA displayed the same general morphology but with smaller dimensions, presenting an average width of 151.17 ± 47.18 nm and an average length of 328.45 ± 88.33 nm. In contrast, ZnO-PS and ZnO-MS consisted predominantly of quasi-spherical particles (Figure 4c,d). ZnO-PS presented an average particle diameter of 66.84 ± 13.28 nm, whereas ZnO-MS exhibited the smallest particle size, with an average diameter of 59.09 ± 11.19 nm.

### Microbiological assay

The antibacterial activity of the synthesized ZnO nanoparticles was evaluated by broth microdilution assays to determine the MIC and MBC against *L. innocua*, *S. aureus*, *E. coli*, and *S. typhimurium* (Table 3; Supporting Information File 1, Figure S1). All synthesized nanoparticles exhibited identical antibacterial activity against *L. innocua*, with bactericidal effects for all materials (MBC/MIC = 4). In contrast, the antibacterial activity against *S. aureus* differed among the synthesized nanoparticles. ZnO-PA exhibited bactericidal activity (MIC 313 µg·mL^−1^; MBC 1,250 µg·mL^−1^), whereas ZnO-PS, ZnO-MA, and ZnO-MS showed bacteriostatic effects with higher MBC/MIC ratios. For *E. coli*, all nanoparticles exhibited the same MIC value (625 μg·mL^−1^); however, ZnO-PA, ZnO-PS and ZnO-MA exhibited bactericidal activity, whereas ZnO-MS exhibited a bacteriostatic effect.

**Table 3:** MIC, MBC, MBC/MIC ratio, and antibacterial effect of the synthesized ZnO nanoparticles against selected bacterial strains.

Bacterium	Sample	MIC (µg·mL^−1^)	MBC (µg·mL^−1^)	MBC/MIC	Effect

*L. innocua*	ZnO-PA	156	625	4	bactericidal
	ZnO-PS	156	625	4	bactericidal
	ZnO-MA	156	625	4	bactericidal
	ZnO-MS	156	625	4	bactericidal
*S. aureus*	ZnO-PA	313	1,250	4	bactericidal
	ZnO-PS	1,250	>20,000	>16	bacteriostatic
	ZnO-MA	625	5,000	8	bacteriostatic
	ZnO-MS	313	10,000	32	bacteriostatic
*E. coli*	ZnO-PA	625	2,500	4	bactericidal
	ZnO-PS	625	2,500	4	bactericidal
	ZnO-MA	625	1,250	2	bactericidal
	ZnO-MS	625	>20,000	>16	bacteriostatic
*S. typhimurium*	ZnO-PA	5,000	10,000	2	bactericidal
	ZnO-PS	2,500	2,500	1	bactericidal
	ZnO-MA	1,250	20,000	16	bacteriostatic
	ZnO-MS	1,250	>20,000	>16	bacteriostatic

For *S. typhimurium*, ZnO-PS and ZnO-PA exhibited bactericidal activity; however, the MIC and MBC values obtained for ZnO-PA (5,000 and 10,000 µg·mL^−1^, respectively) were higher than those determined for ZnO-PS (2,500 µg·mL^−1^ for both MIC and MBC). In contrast, ZnO-MA and ZnO-MS showed bacteriostatic activity. The positive control (amikacin) exhibited MIC values of 10 µg·mL^−1^ for *L. innocua*, 20 µg·mL^−1^ for *S. aureus* and *E. coli*, and 39 µg·mL^−1^ for *S. typhimurium* (Supporting Information File 1, Figure S1).

### Relationships between physicochemical properties and antibacterial activity

**Figure 5:**
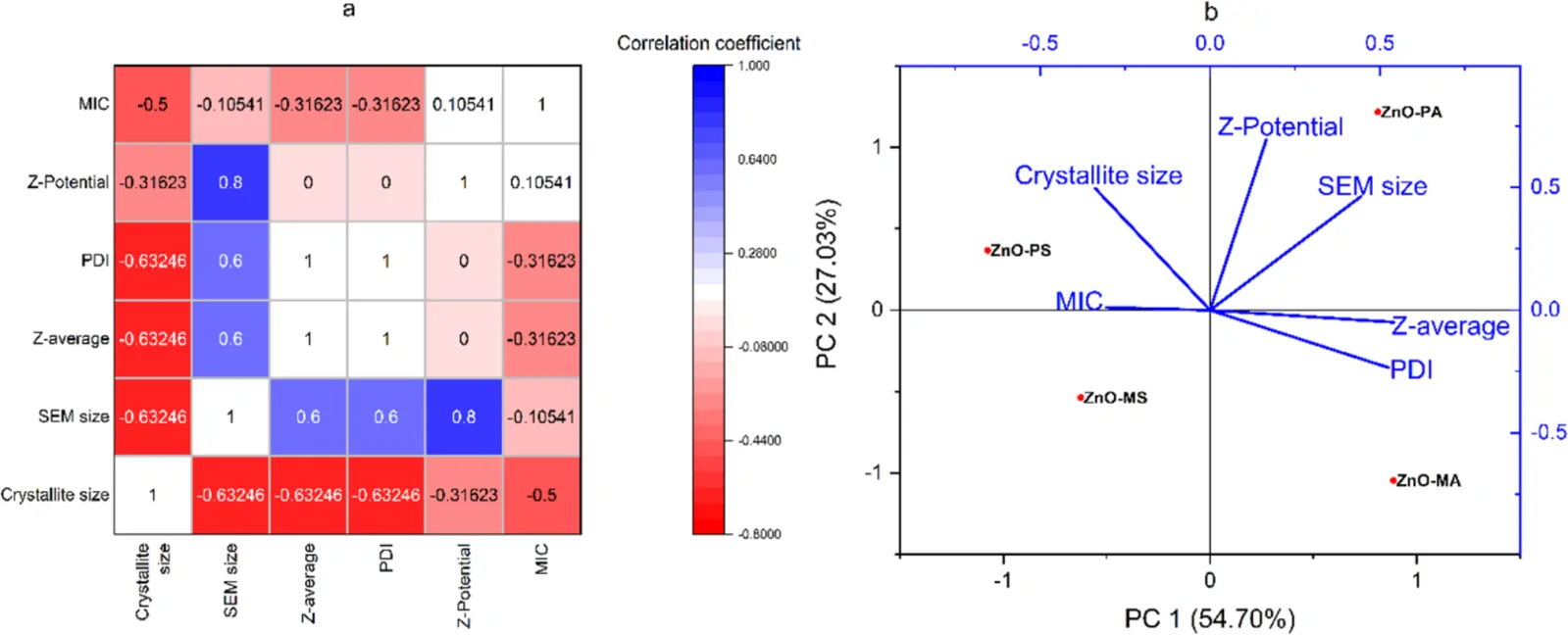
(a) Spearman correlation matrix and (b) PCA of crystallite size, SEM particle size, *Z*-average, PDI, zeta potential, and MIC values for the synthesized ZnO nanoparticles.

To examine the relationships among the physicochemical properties and antibacterial activity of the synthesized ZnO nanoparticles, Spearman rank correlation analysis and principal component analysis (PCA) were performed using crystallite size, SEM particle size, hydrodynamic diameter (*Z*-average), PDI, zeta potential, and MIC values (Figure 5).

The Spearman correlation matrix (Figure 5a) revealed positive and negative associations among the evaluated variables. Crystallite size showed negative correlations with SEM particle size (ρ = −0.63), hydrodynamic diameter (ρ = −0.63), PDI (ρ = −0.63), and MIC (ρ = −0.50). SEM particle size exhibited positive correlations with zeta potential (ρ = 0.80), hydrodynamic diameter (ρ = 0.60), and PDI (ρ = 0.60). A Spearman correlation coefficient of ρ = 1.00 was obtained between hydrodynamic diameter and PDI, whereas zeta potential showed correlation coefficients of ρ = 0.00 with both variables. Principal component analysis explained 81.73% of the total variability, with PC1 accounting for 54.70% and PC2 for 27.03% (Figure 5b). PC1 was mainly associated with hydrodynamic diameter, PDI, SEM particle size, and crystallite size, whereas PC2 was primarily associated with zeta potential. The PCA score plot showed the distribution of the four nanoparticle samples according to their overall physicochemical characteristics.

## Discussion

The plant-assisted synthesis of ZnO nanoparticles is influenced by several experimental variables, including extract composition, pH, temperature, reaction time, precursor concentration, and precipitation chemistry, all of which affect nucleation and crystal growth [18]. The results obtained here indicate that alkaline precipitating chemistry influenced nanoparticle formation without modifying the crystalline phase of ZnO. XRD analysis confirmed that all synthesized nanoparticles crystallized in the hexagonal wurtzite structure (Figure 2a). Although differences in crystallite size were observed among the samples, these differences did not follow a uniform trend according to the precipitating agent, indicating that crystallite development likely reflects the combined influence of precipitation chemistry and plant extract composition.

FTIR spectra of both plant extracts (Figure 1a) showed bands attributed to O–H, C=O, and C–O groups commonly associated with polyphenols and flavonoids, which have been reported to coordinate Zn^2+^ ions and participate in the early stages of ZnO formation through complexation and surface binding [19–21]. Although *P. auritum* and *M. pudica* differed in their total polyphenol and flavonoid contents (Supporting Information File 1, Table S1), both extracts exhibited the same types of oxygen-containing functional groups, with differences mainly reflected in band intensity.

After calcination at 400 °C, the FTIR spectra still retained weak O–H, C=O, and C–O vibrations, suggesting that part of the oxygen-containing species remained chemically associated with the ZnO surface rather than being completely removed during thermal treatment. Similar surface residues have been reported for other biosynthesized ZnO nanoparticles, where strongly adsorbed phytochemical derivatives persist after calcination and contribute to surface chemistry [22–23]. Here, these residual functional groups provide a plausible explanation for the negative surface charge measured for all nanoparticles since oxygen-containing surface species can modify interparticle interactions and influence colloidal behavior [24–25]. Previous phytochemical studies also support this interpretation. *P. auritum* has been reported to contain phenylpropanoids, phenolic acids, and flavonoids [26], whereas *M. pudica* is particularly rich in tannins and other polyphenolic compounds bearing hydroxy and carbonyl groups capable of interacting with Zn^2+^ during nanoparticle formation [27]. Although these phytochemical-derived functional groups contribute to surface functionalization, the results suggest that they do not account for the structural differences observed among the synthesized nanoparticles, which are more consistently explained by the precipitation chemistry.

The role of the alkaline precipitating agent became clearer when the crystallographic parameters were examined. Crystallite sizes calculated using the Scherrer equation closely matched those obtained by the Williamson–Hall approach, indicating that peak broadening was dominated by crystallite size rather than by lattice strain [28]. This interpretation agrees with the low microstrain values determined for all samples and with the nearly constant lattice parameters (*a* = 0.325 nm; *c* = 0.520–0.521 nm), which are in good agreement with those reported for hexagonal wurtzite ZnO [24,29]. These observations indicate that neither NaOH nor NH_4_OH modified the ZnO crystal lattice; instead, their influence was exerted on the crystallization pathway leading to particle formation.

While the crystal structure remained essentially unchanged, differences in crystallite development were observed among the four samples. ZnO-MA exhibited the smallest crystallite size according to both the Scherrer and Williamson–Hall methods (15.35 and 13.76 nm, respectively) and the highest dislocation density, whereas ZnO-PA, ZnO-PS, and ZnO-MS showed comparatively similar crystallite sizes. Therefore, crystallite size cannot be generalized solely as a function of the alkaline precipitating agent. Instead, the particularly small crystallite domains observed for ZnO-MA suggest that crystal development resulted from the combined influence of the precipitation environment and the specific chemical environment generated by the *M. pudica* extract. NaOH and NH_4_OH nevertheless provide distinct precipitation environments: NaOH dissociates completely in aqueous solution and rapidly increases OH^−^ availability, whereas NH_4_OH releases hydroxide ions through the NH_3_/H_2_O/NH_4_^+^ equilibrium and may also promote transient zinc–ammine complex formation [30–32]. These differences can affect nucleation and growth kinetics, but their effect on crystallite size appears to depend on the specific plant extract rather than following a uniform precipitating-agent-dependent trend.

The differences identified at the crystallographic level were also reflected in particle morphology and colloidal behavior. Regardless of the plant extract employed, NaOH consistently produced smaller quasi-spherical particles, whereas NH_4_OH favored the formation of larger rod-like structures. These morphological differences reflect the distinct precipitation environments promoted by each alkaline agent and indicate that precipitation chemistry strongly influences particle growth, morphology, and the organization of crystalline domains into larger particles.

To interpret these results correctly, it is important to distinguish among crystallite size, particle size, and hydrodynamic size since each parameter describes a different structural level of the synthesized nanoparticles. The crystallite size obtained from XRD corresponds to coherently diffracting crystalline domains, whereas FESEM provides the dimensions of dried particles or particle aggregates. DLS, in contrast, measures the hydrodynamic diameter of particles dispersed in suspension, which includes the hydration layer, the electrical double layer, adsorbed surface species, and any aggregation occurring in the colloidal system. Accordingly, the crystallite sizes determined by XRD (15–25 nm) were substantially smaller than the particle dimensions observed by FESEM (60–780 nm), indicating that the synthesized particles are composed of multiple crystalline domains rather than being single crystals. Similar multiscale organization has frequently been reported for biosynthesized ZnO nanoparticles, where primary nanocrystals coalesce during growth and subsequent thermal treatment to form larger polycrystalline particles [22,24].

The colloidal measurements followed the same trend. Nanoparticles synthesized with NaOH exhibited smaller hydrodynamic diameters and narrower size distributions than those obtained with NH_4_OH, whereas the latter showed broader distributions, indicating a greater tendency to aggregate in suspension. This behavior is consistent with the particle morphologies observed by FESEM and agrees with previous observations that elongated particles are more prone to aggregation than quasi-spherical ones [33]. Nevertheless, particle morphology alone cannot account for the hydrodynamic behavior. The relatively low absolute zeta potential values (−9 to −26 mV) indicate only moderate electrostatic stabilization, which favors attractive interactions among particles and contributes to the formation of larger aggregates in aqueous suspension.

The FTIR results provide additional insight into this behavior from a surface chemistry perspective. Even after calcination, weak oxygen-containing functional groups remained associated with the nanoparticle surface, indicating that part of the phytochemical-derived species persisted after thermal treatment [22–23]. These surface groups are likely to contribute to the negative surface charge measured for all samples and to influence interparticle interactions in suspension [24–25]. Although they provide a certain degree of electrostatic stabilization, the measured zeta potential values indicate that this effect is insufficient to prevent aggregation completely. Thus, the dispersion behavior of the synthesized ZnO nanoparticles reflects the combined influence of particle morphology, residual surface chemistry, and electrostatic interactions rather than any single physicochemical property [34].

Considered together, the characterization techniques describe different but complementary aspects of nanoparticle organization. XRD reveals the dimensions of coherent crystalline domains, FESEM shows the morphology of the resulting particles, and DLS reflects their behavior once dispersed in suspension. Viewed in this way, the apparent differences among crystallite size, particle size, and hydrodynamic diameter are expected rather than contradictory. Instead, they illustrate how precipitation chemistry influences structural organization across multiple length scales, from crystal growth to colloidal behavior, providing the physicochemical basis for the biological responses discussed below.

The structural and colloidal differences described above were ultimately reflected in the antibacterial performance of the synthesized ZnO nanoparticles. Although the MIC values did not follow a consistent trend with respect to the alkaline precipitating agent, clearer differences emerged when bactericidal activity was considered. ZnO-PA was the only sample that exhibited bactericidal activity against all four bacterial species evaluated, whereas ZnO-PS, ZnO-MA and ZnO-MS behaved as bacteriostatic agents against at least one microorganism (Table 3). These findings indicate that antibacterial activity cannot be attributed to a single physicochemical property but instead reflects the combined influence of crystallite development, particle morphology, surface chemistry, and colloidal behavior.

The exploratory multivariate analyses support this interpretation. Rather than identifying a single property associated with antibacterial performance, the Spearman correlation matrix and principal component analysis highlighted the interdependence among the evaluated physicochemical variables. Crystallite size, SEM particle size, hydrodynamic diameter, polydispersity index, zeta potential, and antibacterial activity showed interrelated patterns in the multivariate analysis, and no single parameter adequately explained the biological response. Given that only four ZnO nanoparticle samples were included, these analyses should be regarded as exploratory rather than predictive. Instead, they provide a useful visualization of the relationships among structural, colloidal, and biological characteristics and complement the experimental observations discussed above.

Several trends identified by the multivariate analyses were fully consistent with the physicochemical characterization. An inverse trend was observed between crystallite size and both SEM particle size and hydrodynamic diameter, indicating that nanoparticles composed of smaller crystalline domains did not necessarily produce smaller particles or colloidal aggregates. Likewise, hydrodynamic diameter and PDI exhibited identical rankings (ρ = 1.00), showing that larger aggregates were consistently associated with broader size distributions in suspension. The PCA also distributed the samples according to their combined structural and colloidal characteristics: ZnO-PA and ZnO-MA were positioned on the positive side of PC1 associated mainly with larger SEM particle size, hydrodynamic diameter, and PDI, whereas ZnO-PS and ZnO-MS were positioned toward the opposite side, consistent with their smaller particle dimensions and lower aggregation in suspension. Crystallite size did not follow this same sample distribution, as ZnO-MA exhibited the smallest crystallite size among the four materials. Therefore, the PCA separation should be interpreted as reflecting the combined contribution of multiple physicochemical variables rather than a direct relationship with crystallite size.

One aspect deserves particular attention. Despite contributing to a negatively charged surface, the residual phytochemical-derived functional groups did not translate into a direct relationship between zeta potential and antibacterial activity. Likewise, the superior bactericidal performance of ZnO-PA, despite its comparatively larger hydrodynamic diameter, indicates that particle aggregation by itself is insufficient to explain the biological response. Instead, the results point toward a combined effect of particle morphology, surface chemistry, and colloidal organization, all of which determine the initial interactions between nanoparticles and bacterial cells.

The experiments performed here do not allow the molecular mechanism of bacterial inactivation to be established directly. However, numerous studies have shown that ZnO nanoparticles exert their antimicrobial activity through multiple concurrent processes rather than through a single mechanism [35]. According to previous studies, the initial interactions occur at the nano–bio interface, where particle morphology, surface chemistry, surface charge, and aggregation state influence adhesion to the bacterial envelope and affect membrane integrity [20,36]. Following this initial contact, ROS generation has frequently been proposed as one of the main contributors to bacterial inactivation because oxidative stress damages membrane lipids, proteins, and nucleic acids [37]. In parallel, partial dissolution of ZnO may release Zn^2+^ ions that interfere with intracellular metal homeostasis and enzyme activity, although the relative contribution of this pathway depends strongly on physicochemical properties of the nanoparticles and on the surrounding medium [6,14,37]. Because ROS production, Zn^2+^ release, and membrane damage were not evaluated experimentally in this work, these mechanisms are discussed only as literature-supported explanations for the observed antibacterial activity.

**Figure 6:**
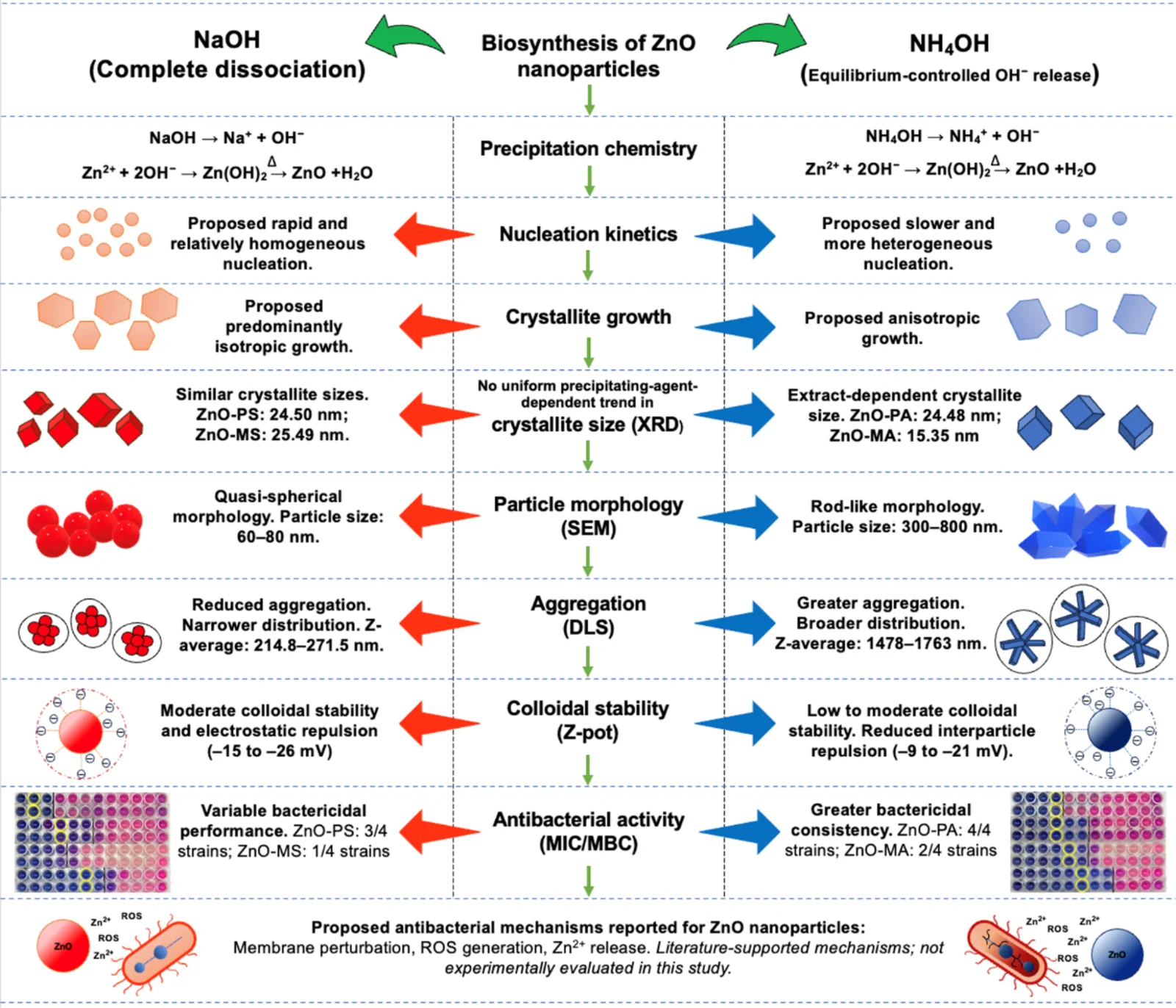
Proposed mechanistic framework illustrating the influence of precipitation chemistry on nucleation, crystal growth, surface functionalization, colloidal stability, and antibacterial performance of biosynthesized ZnO nanoparticles synthesized using *P. auritum* and *M. pudica* extracts.

Overall, the results indicate that antibacterial performance arises from the combined effects of precipitation chemistry, crystal growth, particle morphology, surface functionalization, and colloidal behavior. Rather than acting independently, these properties collectively shape the nano–bio interface that ultimately governs the interaction between ZnO nanoparticles and bacterial cells. This interpretation also provides the conceptual basis for the mechanistic model proposed in Figure 6.

The results presented throughout this study support an integrated mechanistic framework in which precipitation chemistry governs the structural evolution and, ultimately, the antibacterial performance of biosynthesized ZnO nanoparticles (Figure 6). Rather than modifying the intrinsic crystal structure of ZnO, the alkaline precipitating agent regulates hydroxide ion availability during synthesis, thereby controlling nucleation kinetics and subsequent crystal growth.

Rapid OH^−^ generation by NaOH creates high supersaturation, promoting the simultaneous formation of numerous nuclei and relatively homogeneous crystal growth [38]. In contrast, NH_4_OH releases hydroxide ions gradually through the NH_3_/H_2_O/NH_4_^+^ equilibrium while transient zinc–ammine complexes may also form in solution [39–40]. Under these conditions, nucleation proceeds more slowly and favors anisotropic crystal growth without altering the hexagonal wurtzite structure of ZnO. These differences are reflected in crystallite size, particle morphology, and colloidal behavior, all of which are consistent with the physicochemical characterization presented above.

Surface chemistry represents a second level of control within this framework. FTIR analysis showed that oxygen-containing functional groups derived from the plant extracts remained partially associated with the nanoparticle surface after calcination. These residual surface species are likely to contribute to the negative surface charge measured by zeta potential analysis and influence particle–particle interactions in suspension. Consequently, nanoparticle architecture is determined by the combined effects of precipitation chemistry and surface functionalization rather than by either factor alone.

The observed antibacterial response emerges from this physicochemical architecture. Variations in crystallite development, particle morphology, colloidal organization, and surface chemistry determine the initial interactions between nanoparticles and bacterial cells. According to previous reports, possible antibacterial pathways for ZnO include membrane perturbation, ROS generation, and the contribution of dissolved Zn^2+^ ions [6,14,20,36–37]; however, none of these mechanisms was experimentally evaluated in the present study. They are therefore discussed only as literature-supported mechanisms that may help contextualize the observed antibacterial response and should not be interpreted as mechanisms demonstrated by our results.

Figure 6 integrates this sequence of events, bringing together the experimental evidence obtained from XRD, FTIR, FESEM, DLS, zeta potential measurements, antibacterial assays, and exploratory multivariate analyses into a single conceptual model. Rather than representing independent observations, these techniques describe successive stages in nanoparticle development, from hydroxide ion availability during synthesis to the establishment of the nano–bio interface responsible for antibacterial activity. Viewed in this context, precipitation chemistry emerges as a key design parameter for tuning the physicochemical properties and biological performance of biosynthesized ZnO nanoparticles.

## Conclusion

This study demonstrates that the physicochemical and antimicrobial properties of ZnO nanoparticles synthesized using *P. auritum* and *M. pudica* extracts are strongly influenced by the precipitating agent used during synthesis. While both systems produced crystalline ZnO with retained surface functionalization from plant-derived phytochemicals, the alkaline precipitating agent played a decisive role in determining nanoparticle morphology and colloidal stability. Nanoparticles ZnO-PS and ZnO-MS exhibited more uniform quasi-spherical morphologies and narrower hydrodynamic size distributions, whereas NH_4_OH promoted anisotropic structures consistent with the proposed gradual hydroxide release and transient zinc–ammine complex formation during nucleation. These structural differences were reflected in the antibacterial performance of the materials, with ZnO-PA showing bactericidal activity against all tested bacterial strains. Overall, these findings support the proposed mechanistic framework and identify precipitation chemistry as a key design parameter for tailoring the structural, colloidal, and antibacterial properties of plant-mediated ZnO nanoparticles intended for antimicrobial applications.

## Experimental

### Materials

Leaves of *P. auritum* and *M. pudica* were collected in Tabasco, Mexico (17°15′–18°39′ N, 91°00′–94°17′ W). Zinc acetate dihydrate (Zn(CH_3_COO)_2_·2H_2_O, ≥98%), gallic acid (≥97%), Folin–Ciocalteu reagent (1 N), quercetin hydrate (≥95%), and resazurin sodium salt were purchased from Sigma-Aldrich (St. Louis, MO, USA). Ammonium hydroxide (NH_4_OH, 28–30% NH_3_, ACS reagent) was obtained from Meyer (Mexico City), sodium hydroxide (NaOH, ≥97%) and methanol (≥99.8%) from Merck (Darmstadt, Germany), and ethanol (96%, v/v) from Alcoholera Zapopan (Guadalajara, Mexico). The bacterial strains *Listeria innocua* ATCC 33090, *Staphylococcus aureus* ATCC 6538, *Escherichia coli* ATCC 25922, and *Salmonella typhimurium* ATCC 14028 were used for the antibacterial assays. Tryptic soy broth (TSB) and tryptic soy agar (TSA) were obtained from Bioxon BD (State of Mexico, Mexico). Antibacterial susceptibility assays were performed using sterile 96-well microplates (Nunc F96, Thermo Fisher Scientific, Waltham, MA, USA) and amikacin (2,500 μg·mL^−1^; Laboratorios AMSA, Mexico City, Mexico).

### Preparation of aqueous plant extracts

Aqueous extracts of *P. auritum* and *M. pudica* were prepared following the method described by Sharma et al. [41] with slight modifications. Briefly, 50 g of fresh leaves were mixed with 500 mL of deionized water and heated at 60 °C for 2 h. The suspension was then allowed to cool to room temperature. To enhance the release of plant metabolites, the extract was homogenized for 30 s using an immersion blender, filtered through Whatman No. 1 filter paper, and stored at 4 °C until further use.

### Phytochemical analysis of aqueous extracts

The functional groups present in the aqueous extracts were analyzed by FTIR using a Nicolet iS50 spectrophotometer (Thermo Fisher Scientific, Waltham, MA, USA) equipped with an attenuated total reflectance (ATR) accessory. Spectra were recorded over the range of 4000–400 cm^−1^ using 32 scans. Total polyphenol and flavonoid contents were determined using the Folin–Ciocalteu and aluminum chloride colorimetric methods, respectively, following Torres-López et al. [42] and Zhu et al. [43]. Gallic acid and quercetin were used as calibration standards, and all measurements were performed in triplicate.

### Biosynthesis of ZnO nanoparticles

**Figure 7:**
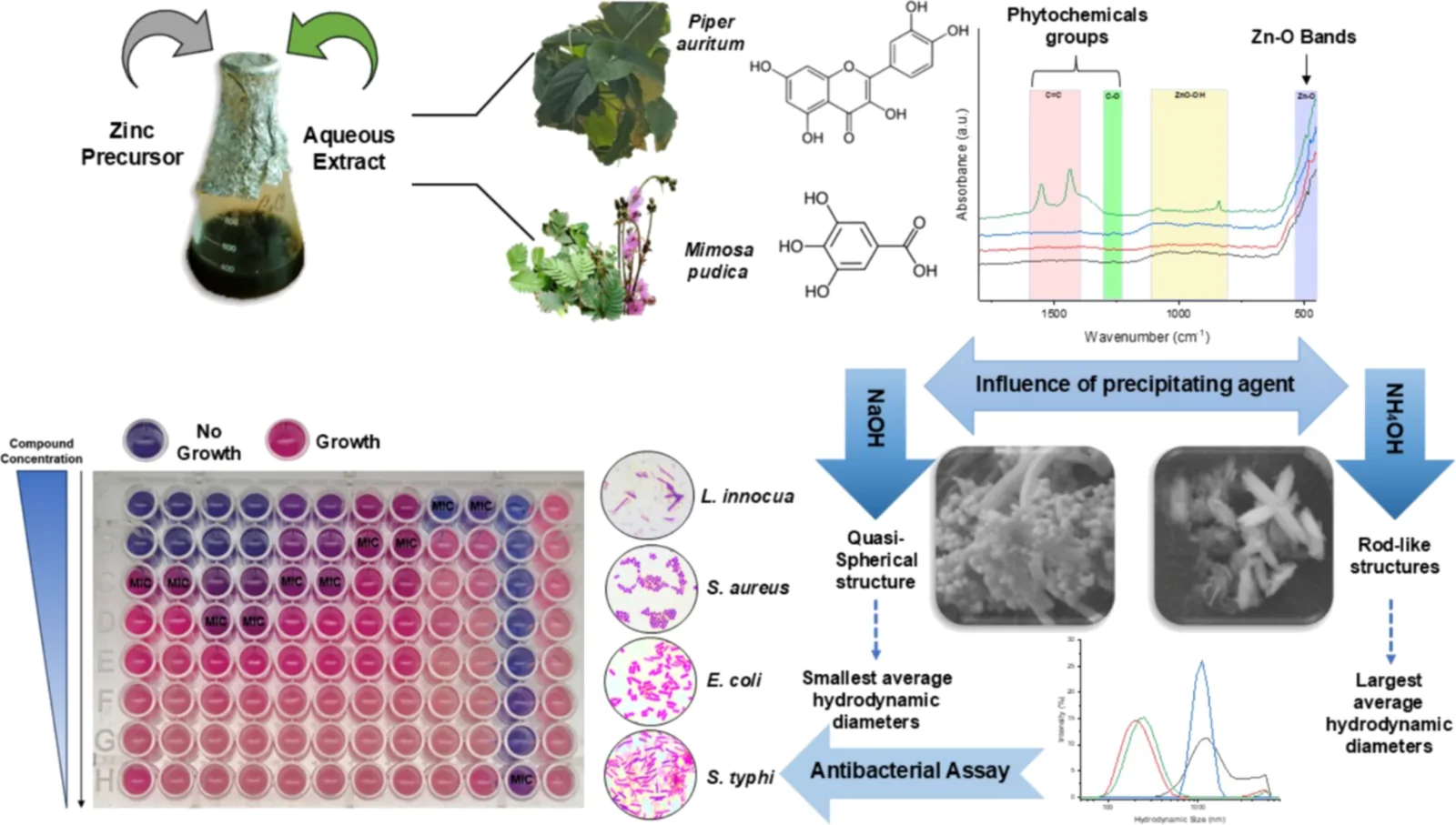
Schematic representation of the experimental workflow, including the preparation of aqueous plant extracts, biosynthesis of ZnO nanoparticles using different precipitating agents, physicochemical characterization, and antibacterial evaluation.

For the synthesis of ZnO nanoparticles, 180 mL of zinc acetate dihydrate solution (0.5 M) was heated to 65 ± 5 °C. Subsequently, 45 mL of the corresponding aqueous plant extract was added dropwise under constant stirring, and the reaction mixture was maintained for 1 h. After cooling to room temperature, the pH was adjusted to 10 using either NH_4_OH (2 M) or NaOH (2 M). The resulting suspensions were centrifuged at 4,500 rpm for 30 min, and the precipitates were washed three times with distilled water followed by a final wash with 96% ethanol. The precipitates were dried at 70 °C for 8 h and subsequently calcined at 400 °C for 2 h using a heating rate of 5 °C·min^−1^. The samples were labeled according to the plant extract and the alkaline precipitating agent used during synthesis (Table 1). A schematic representation of the experimental setup is shown in Figure 7.

### Characterization of ZnO nanoparticles

FTIR spectra of the calcined ZnO nanoparticles were recorded under the same analytical conditions described for the aqueous plant extracts. The crystalline structure of the samples was examined by XRD using a D8 Advance diffractometer (Bruker, Billerica, MA, USA) equipped with Cu Kα radiation (λ = 1.5406 Å) operating at 40 kV and 25 mA. Diffraction patterns were collected over a 2θ range of 20–80°, with a step size of 0.02° and a counting time of 0.3 s per step.

Hydrodynamic size and zeta potential were determined using DLS with a Zetasizer Nano ZS (Malvern Instruments Ltd., Worcestershire, UK) equipped with a 633 nm laser and a detector positioned at 173°. ZnO nanoparticles were dispersed in distilled water at a concentration of 10 µg·mL^−1^ and sonicated for 15 min using an ultrasonic cleaner (Cole-Parmer 8891R-MT, 42 kHz). Measurements were performed at 25 °C. Zeta potential measurements were carried out using the same instrument equipped with a ZEN1002-Zeta Dip CELL electrode. Morphology and particle size of the nanoparticles were examined by FESEM (ZEISS Gemini SEM 300, Carl Zeiss Microscopy GmbH, Oberkochen, Germany) operated at an accelerating voltage of 1–15 kV. Prior to analysis, the samples were mounted on carbon adhesive tape and sputter-coated with a thin Au–Pd layer. Particle size measurements were performed using ImageJ v1.54 (National Institutes of Health, Bethesda, MD, USA). The mean particle size and standard deviation were calculated from measurements of 50 individual particles (*n* = 50) for each sample.

### X-ray diffraction analysis

Crystallite size was estimated using the Scherrer equation (Equation 1):


[1]

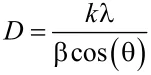



where *k* is the shape factor (Scherrer constant), λ is the X-ray wavelength (1.5406 Å), β represents the full width at half maximum (FWHM, in radians), and θ is the Bragg diffraction angle. To evaluate the contribution of lattice strain to diffraction peak broadening, the Williamson–Hall (W–H) method was applied according to the uniform deformation model. The total peak broadening (β_T_) was considered as the sum of crystallite size broadening (β_D_) and strain-induced broadening (β_ε_) (Equation 2).


[2]

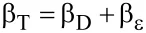



The Williamson–Hall relationship is given by Equation 3:


[3]

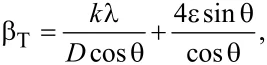



where ε represents the lattice microstrain and *D* is the crystallite size.

The dislocation density (δ) was estimated using Equation 4:


[4]

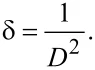



The lattice parameters (*a* and c) of the hexagonal wurtzite structure were calculated from the indexed diffraction peaks using the corresponding crystallographic equations.

### Microbiological assay

The MIC and MBC were determined following the Clinical and Laboratory Standards Institute (CLSI M07) [44] protocol with minor modifications. Serial twofold dilutions of ZnO nanoparticles ranging from 20,000 to 9.8 µg·mL^−1^ were prepared in sterile 96-well plates (Nunc F96 microtiter plates, Thermo Fisher Scientific, Waltham, MA, USA). Each well contained 100 µL of diluted nanoparticles and 100 µL of bacterial suspension adjusted to 1 × 10^7^ CFU·mL^−1^ in tryptic soy broth. Sterile distilled water and amikacin (2,500 µg·mL^−1^) were used as controls. MIC values were determined after incubation for 24 h at 37 °C by adding 30 µL of 0.01% (w/v) resazurin solution. The appearance of a pink color (resorufin formation) indicated the presence of viable bacteria, whereas wells remaining blue were considered to show inhibition of bacterial growth. MBC values were determined by subculturing 10 µL from each well onto tryptic soy agar plates. No visible bacterial growth after incubation (24 h at 37 °C) signaled loss of bacterial viability. The antibacterial effect was classified by the MBC/MIC ratio, where values ≤4 were considered bactericidal and values >4 bacteriostatic.

### Correlation and principal component analyses

Relationships among the physicochemical properties and antibacterial activity of the synthesized ZnO nanoparticles were explored using Spearman rank correlation analysis and PCA. The evaluated variables included crystallite size (Scherrer), SEM particle size, *Z*-average, PDI, zeta potential, and MIC values. Correlation coefficients were calculated using Spearman’s rank method. The analyses were performed using the four synthesized ZnO nanoparticle samples. Prior to PCA, the variables were standardized to zero mean and unit variance. Correlation matrices and PCA biplots were generated using OriginPro 2021 (OriginLab Corporation, Northampton, MA, USA). Because the dataset consisted of only four samples, these multivariate analyses were used exclusively as exploratory tools to visualize relationships among variables rather than for inferential statistical analysis.

## Supporting Information

File 1 Total polyphenol and flavonoid contents of the plant extracts and representative microdilution assay images.

## Data Availability

All data that supports the findings of this study is available in the published article and/or the supporting information of this article.
