# Supplementary material for: Effect of alkaline precipitation chemistry on morphology, colloidal stability, and antibacterial activity of ZnO nanoparticles synthesized using plant extracts
Source: Beilstein J Nanotechnol. 2026 Oct 21;17:1438–52. doi: 10.3762/bjnano.17.99 (PMC13698874; doi:10.3762/bjnano.17.99)
Supplement: Supplementary file 1 [file Beilstein_J_Nanotechnol-17-1438-s001.pdf]

BEILSTEIN JOURNAL OF NANOTECHNOLOGY

Supporting Information

for

Effect of alkaline precipitation chemistry on morphology, colloidal stability, and antibacterial activity of ZnO nanoparticles synthesized using plant extracts

Fanny Jauregui González, Dora María Frías Márquez, Susana de la Rosa García, Cinthia García Mendoza, Rosendo López González, Mayra A. Álvarez Lemus and Sergio Gómez Cornelio

Beilstein J. Nanotechnol. **2026,** *17,* 1438–1452. doi:10.3762/bjnano.17.99

Total polyphenol and flavonoid contents of the plant extracts and representative microdilution assay images

License and Terms: This is a supporting information file under the terms of the Creative Commons Attribution License (https://creativecommons.org/licenses/by/4.0). Please note that the reuse, redistribution and reproduction in particular requires that the author(s) and source are credited and that individual graphics may be subject to special legal provisions.
The license is subject to the *Beilstein Journal of Nanotechnology* terms and conditions: (https://www.beilstein-journals.org/bjnano/terms)

Table S1: Total polyphenol and flavonoid contents in the aqueous extracts of *P. auritum* and *M. pudica*. Polyphenol content is expressed as mg of gallic acid equivalents (GAE) per g of extract, and flavonoid content as mg of quercetin equivalents (QE) per g of extract.

**Plant extract**	**Polyphenols (mg GAE g^{-1} extract)**	**Flavonoids (mg QE g^{-1} extract)**
Piper auritum	6.74 ± 0.51	6.19 ± 0.08
Mimosa pudica	16.62 ± 0.24	10.1 ± 0.09

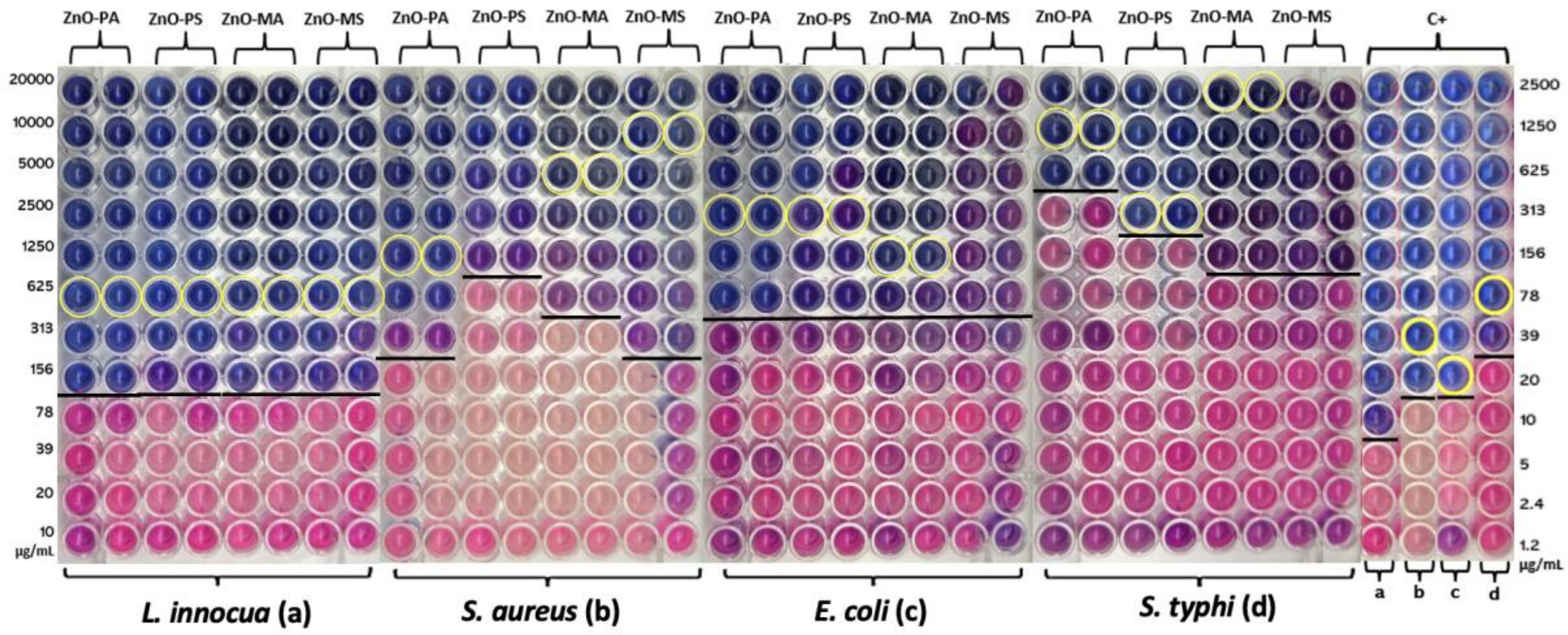

Figure S1: Broth microdilution assay used to determine the antibacterial activity of the synthesized ZnO nanoparticles against *L. innocua* (a), *S. aureus* (b), *E. coli* (c) and *S. typhimurium* (d). The black line indicates the minimum inhibitory concentration (MIC), while the yellow circle indicates the minimum bactericidal concentration (MBC). Amikacin was used as the positive control (C+).
